# The Role of NLRP3 Inflammasome in Pneumococcal Infections

**DOI:** 10.3389/fimmu.2020.614801

**Published:** 2020-12-14

**Authors:** Surabhi Surabhi, Fabian Cuypers, Sven Hammerschmidt, Nikolai Siemens

**Affiliations:** Department of Molecular Genetics and Infection Biology, University of Greifswald, Greifswald, Germany

**Keywords:** nucleotide-binding and oligomerization domain-like receptors and pyrin domain containing receptor 3, inflammasome, pneumococcus (*Streptococcus pneumoniae*), respiratory infection, immune response

## Abstract

Inflammasomes are innate immune sensors that regulate caspase-1 mediated inflammation in response to environmental, host- and pathogen-derived factors. The NLRP3 inflammasome is highly versatile as it is activated by a diverse range of stimuli. However, excessive or chronic inflammasome activation and subsequent interleukin-1β (IL-1β) release are implicated in the pathogenesis of various autoimmune diseases such as rheumatoid arthritis, inflammatory bowel disease, and diabetes. Accordingly, inflammasome inhibitor therapy has a therapeutic benefit in these diseases. In contrast, NLRP3 inflammasome is an important defense mechanism against microbial infections. IL-1β antagonizes bacterial invasion and dissemination. Unfortunately, patients receiving IL-1β or inflammasome inhibitors are reported to be at a disproportionate risk to experience invasive bacterial infections including pneumococcal infections. Pneumococci are typical colonizers of immunocompromised individuals and a leading cause of community-acquired pneumonia worldwide. Here, we summarize the current limited knowledge of inflammasome activation in pneumococcal infections of the respiratory tract and how inflammasome inhibition may benefit these infections in immunocompromised patients.

## Introduction

The human innate immunity axis plays a pivotal role in detection of pathogen- or damage-associated molecular patterns (PAMPs and DAMPs) and contributes to a crucial inflammatory response. To sense PAMPs and DAMPs, innate immune cells express pattern recognition receptors (PRRs). PRRs are classified into five families: Toll-like receptors (TLRs), Nucleotide-binding and oligomerization domain (NOD)-like receptors (NLRs), Retinoic acid-inducible gene (RIG)-I-like receptors, C-type lectin receptors, and Absent in melanoma 2 (AIM2)-like receptor (ALR) (1). Furthermore, other molecules such as cyclic GMP-AMP synthase can sense pathogen-derived DNA (2). Inflammasomes are one of the most recently discovered classes of NLRs (3).

To date, 22 human NLRs are described. Among them, NLR and pyrin domain containing receptor 3 (NLRP3) is by far the best characterized (4). A wide range of stimuli including bacterial pore forming toxins can activate the NLRP3 inflammasome (5). The subsequent release of interleukins (IL) IL-1β and IL-18 induces a diverse range of protective host pathways aiming to eradicate the pathogen (6). However, uncontrolled and excessive hyper-inflammation can be a driver of several inflammatory and autoimmune diseases (7, 8). Implication of the NLRP3 inflammasome in inflammatory diseases has provided new avenues for designing drugs which target the inflammasome and its signaling cascade. However, it is observed that patients who receive NLRP3 or IL-1β inhibitors are disproportionately susceptible to bacterial infections (9). Therefore, it is of high importance to understand the role of NLRP3 inflammasome in bacterial pathogenesis.

## Canonical NLRP3 Inflammasome Activation

NLRP3 inflammasome is a multi-protein complex comprising of a sensor NLRP3 protein, an adaptor apoptosis-associated speck-like protein (ASC), and the zymogen procaspase-1 (10). The cytosolic NLRP3 protein contains an N-terminal Pyrin domain (PYD), a central NACHT domain, and a C-terminal leucine-rich repeat (LRR) domain. The NACHT domain possesses adenosine triphosphatase (ATPase) activity and comprises of nucleotide-binding domain (NBD), helical domain 1 (HD1), winged helix domain (WHD) and helical domain 2 (HD2) (11). The ASC domain is a bipartite molecule that contains an N-terminal PYD domain and a C-terminal caspase activation and recruitment domain (CARD). Procaspase-1 consists of an N-terminal CARD, a central large catalytic p20 subunit, and a C-terminal small catalytic p10 subunit (12).

**Figure 1:**
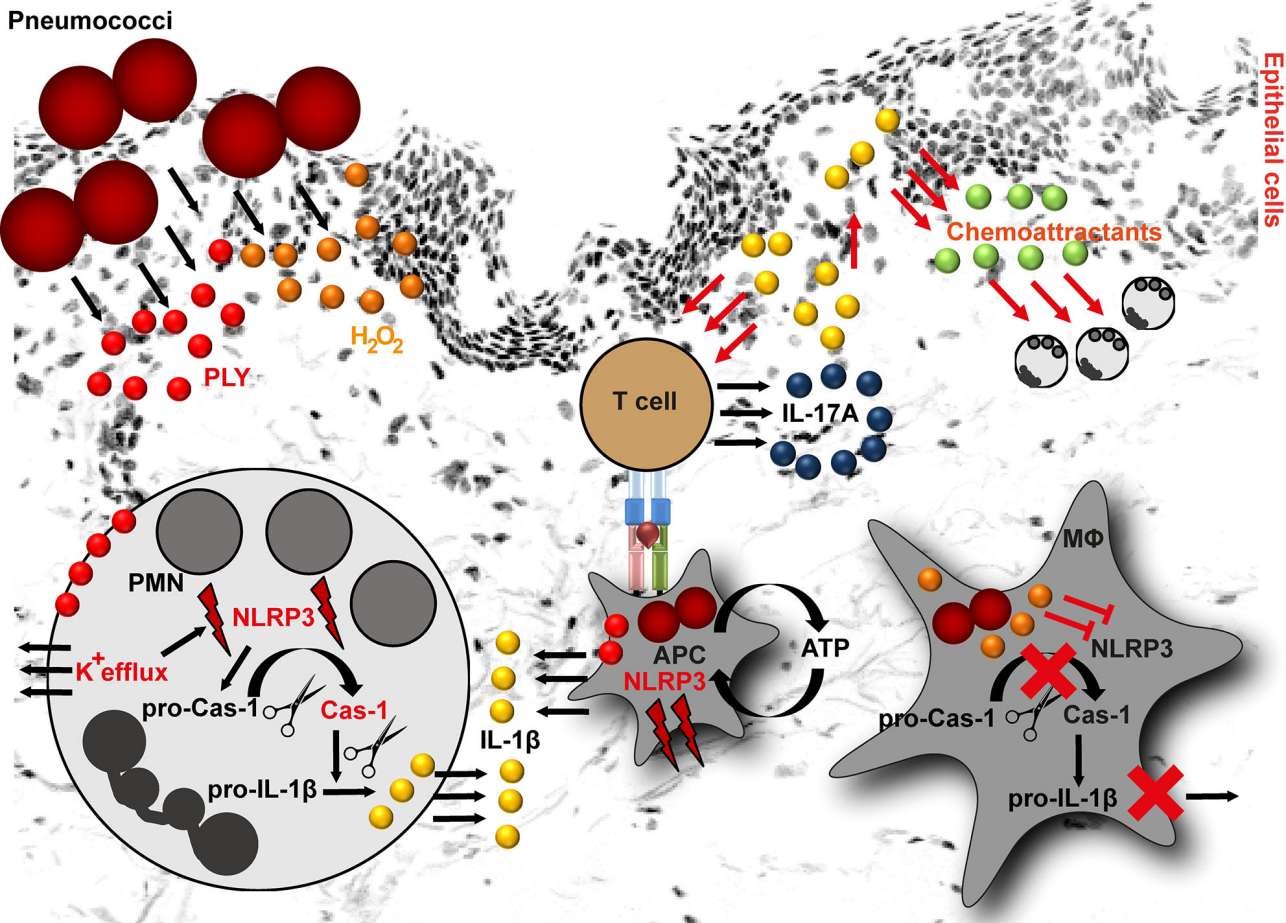
NLRP3 inflammasome activation by pneumococci. Pneumococci secrete two major virulence determinants: pneumolysin (PLY) and hydrogen peroxide (H_2_O_2_). In neutrophils (PMN), PLY-mediated NLRP3 activation is a result of K^+^ efflux. K^+^ efflux activates NLRP3 inflammasome resulting in caspase-1 activation and subsequent cleavage of pro-IL-1β into mature form. In macrophages, PLY-mediated NLRP3 inflammasome activation is among others dependent on ATP. The released IL-1β stimulates epithelial cells. As a result, they release chemoattractants, including CXCL1 and CXCL2. Both chemokines are involved in processes resulting in neutrophil influx. Furthermore, IL-1β is involved in T helper type 17 cells differentiation and subsequent IL-17A release. In contrast to PLY, H_2_O_2_ suppresses NLRP3 inflammasome in macrophages (MΦ) resulting in pro-IL-1β accumulation in these cells (APC, antigen presenting cell).

In resting macrophages, the NLRP3 and pro-IL-1β concentrations are insufficient to initiate activation of the inflammasome (13). Therefore, the NLRP3 inflammasome is activated in a two-step process. The first, so called priming step, is initiated *via* the inflammatory stimuli which are detected by TLRs, tumor necrosis factor receptors (TNFR) or IL-1R. These actions activate downstream the transcription factor NF-κB. NF-κB, in turn, upregulates the expression of NLRP3 and pro-IL-1β. In contrast, the priming step does not affect the expression of ASC, procaspase-1 or IL-18 (14–16). Following priming, a second activation step is essential for the assembly of the inflammasome. NLPR3 is highly diverse in nature and a wide range of stimuli can activate it. Common activators of the NLRP3 inflammasome are pathogens (17), extracellular ATP (18), pathogen associated RNA, proteins and toxins (5, 19, 20), heme (21), endogenous factors (amyloid-β, cholesterol crystals, uric acid crystals) (22–24), and environmental factors (silica and aluminum salts) (24, 25). These activators do not directly interact with the inflammasome but rather cause various changes at the cellular level. These include changes in cell volume (26), ionic fluxes (27), lysosomal damage (28), ROS production, and mitochondrial dysfunction (29). The second activation step is essential for cells such as macrophages and epithelial cells. In contrast, human monocytes can release mature IL-1β already after priming (30, 31). Upon activation, oligomerization of the NLRP3 complex occurs *via* homotypic PYD-PYD interaction of the sensor and adaptor protein, and CARD-CARD interaction of the adaptor and procaspase-1 (**Figure 1**). Following assembly, recruited procaspase-1 is converted to bioactive caspase-1 through proximity induced auto-proteolytic cleavage (32). Subsequently, caspase-1 cleaves the cytokine precursors pro-IL-1β and pro-IL-18 into mature forms. Simultaneously, caspase-1 cleaves gasdermin-D (GSDMD). After proteolytic cleavage, the C-terminal GSDMD (GSDMD-C) remains in the cytosol, while GSDMD-N anchors the cell membrane lipid. The lipid binding allows GSDMD-N to enter the lipid bilayer. Subsequent GSDMD-N oligomerization within the membrane results in pore formation leading to cell swelling and lysis. The pores serve thereby as protein secretion channels for IL-1β and IL-18. This form of a programmed inflammatory cell death is called pyroptosis (33–35). However, lytic GSDMD-N dependent secretion of IL-1β does not apply universally to all cell types. Studies on neutrophils have shown that GSDMD-N does not localize at the plasma membrane. Instead, it co-localizes with membranes of azurophilic granules and LC3^+^ autophagosomes resulting in a non-lytic pathway dependent IL-1β secretion which depends on autophagy machinery (36). Alongside with IL-1β, other pro-inflammatory cytokines, eicosanoids, and alarmins are released into the extracellular space. These actions accentuate the inflammatory state by recruiting additional inflammatory immune cells of different lineages (37–39).

Apart from the canonical, non-canonical NLRP3 inflammasome activation is described (40). Non-canonical inflammasome activation is triggered by caspases-4/5 in humans (41). However, the noncanonical form can sense only Gram-negative bacteria. Therefore, it potentially does not play a role in Gram-positive bacterial infections (40).

## Approved IL-1β Inhibiting Drugs and Their Side-Effects in Patients

NLRP3 inflammasome signaling is implicated in the onset of a number of diseases, including gout (24), atherosclerosis (23), type ІІ diabetes (42, 43), Cryopyrin-associated periodic syndrome (CAPS) (44), various types of cancer (45), and inflammatory bowel disease (IBD) (46). In the following, we give just two examples of the role of NLRP3 in auto-inflammatory and auto-immune diseases.

CAPS summarizes three auto-inflammatory diseases caused by mutations in the *NLRP3* gene. These include familial cold auto-inflammatory syndrome (FCAS), Muckle-Wells syndrome (MWS), and neonatal onset multisystem inflammatory disease (NOMID). In most cases, CAPS manifests during the childhood and is characterized by spontaneous NLRP3 activation and excessive IL-1β production resulting in frequent episodes of fever, skin rashes, joint and eye inflammation. In severe cases, children can suffer from periorbital edema, amyloidosis, polyarthralgia, growth retardation, and death (47, 48). *In vivo* studies with transgenic mice expressing the human disease-associated R258W (MWS) or A350V and L351P (FCAS) mutations in the *NLRP3* gene demonstrated the detrimental role of IL-1β in these diseases (49, 50). Genetic deletion of the IL-1R efficiently rescued *NLRP3^A350V^* and partially *NLRP3^L351P^* mice from neonatal lethality (49).

Rheumatoid arthritis (RA) is a chronic autoimmune disease characterized by persistent synovial inflammation and hyperplasia of the diarthrodial joints and progressive destruction of cartilage and bone (51). In particular, chondrocytes- and monocytes-derived TNF and IL-1β are associated with hyper-inflammatory processes in affected joints (52). At the local level, even low concentrations of IL-1β induce production and secretion of matrix metalloproteinases, which are mainly involved in destructive processes (53). Furthermore, IL‐1β assists in T helper type 17 (Th17) cells differentiation and subsequent IL-17A production. Both processes further contribute to the hyper-inflammatory state of RA (54). These data implicate NLRP3 inflammasome as one of the contributing factors to RA progression. In line with this, several studies have shown that *NLRP3* and other inflammasome-related genes are highly upregulated in monocytes, macrophages, and dendritic cells of RA patients. Furthermore, *NLRP3* gene polymorphisms *(e.g.*, rs35829419, rs10754558, rs4612666) were associated with RA manifestation and pathogenesis [reviewed in (55)].

Although the above mentioned diseases affect different organs and are diverse in nature, they are also characterized by a common feature, namely elevated levels of IL-1β. It is of significance to mention that IL-1β release is not limited to NLRP3 inflammasome activation. A variety of mechanisms, including AIM2 inflammasome activation, which also plays a crucial role in bacterial detection, can result in production and release of IL-1β [reviewed in (56)]. Therefore, treatments target various components of the signaling cascade and particularly IL-1. Several strategies that combat IL-1 action have undergone substantial clinical trials. Some of them are summarized in **Table 1**. Anakinra, Rilonacept, and Canakinumab are clinically approved IL-1 inhibiting drugs and the best studied agents (73). Anakinra is an IL-1 receptor antagonist and is used among others for the treatment of RA, acute gouty arthritis, and CAPS. It blocks the action of both IL-1α and IL-1β (74–76). Clinical trials with Anakinra reported elevated numbers of infectious episodes in Anakinra-treated patients as compared to the placebo-treated group during the first 6 months of treatment. Furthermore, the incidence of serious infections was increased. These infections comprised mainly of cellulitis, pneumonia, and bone and joint infections as well (57). Rilonacept is a dimeric fusion protein that contains two IL-1 receptors attached to the Fc portion of human IgG1. Similar to Anakinra, it blocks the activity of IL-1 isoforms but does not interact with the IL-1 receptor. Rilonacept is used to treat CAPS and the most reported side-effects include skin reactions and upper respiratory tract infections (58). Canakinumab is a monoclonal IgG1 that specifically targets IL-1β and is commonly used for treatment of Periodic Fever Syndromes, MWS, and acute gouty arthritis (59, 60). Among the various side effects, respiratory tract infections were the most reported side effect in clinical trials (60).

**Table 1:** NLRP3 inflammasome inhibitors used in clinics.

Drug	Target	Inhibition mechanism	Treatment	Reference
Anakinra	IL-1 receptor	IL-1 receptor antagonist	Rheumatoid arthritis, Cryopyrin-associated periodic syndrome	(57)
Rilonacept	IL-1α and IL-1β	IL-1 blocker	Cryopyrin-associated periodic syndrome	(58, 59)
Canakinumab	IL-1β	Monoclonal IgG1 antibody	CAPS and other Periodic Fever Syndromes, active Still’s disease	(59–61)
Tranilast^*^	NACHT domain	Inhibits the NLRP3 oligomerization	Bronchial asthma, atypical dermatitis, allergic conjunctivitis, keloids, and hypertrophic scar	(62)
Gevokizumab^#^	IL-1β	Monoclonal anti-IL-1β antibody	Diabetes, autoimmune disease	(63, 64)
LY2189102^#^	IL-1β	Humanized monoclonal anti-IL-1β antibody	Rheumatoid arthritis, Type 2 diabetes	(65)
Glyburide^#^	ATP-sensitive K^+^ channels	Indirect inhibition of the NLRP3 inflammasome	Type 2 diabetes, gestational diabetes	(66–68)
VX-740^#^ (Pralnacasan)	Caspase-1	Non-peptide caspase-1 inhibitor	Osteoarthritis and rheumatoid arthritis	(69)
VX-765^#^ (Belnacasan)	Caspase-1 Caspase-4	Peptidomimetic metaboliteCaspase-1/4 inhibitor	Rheumatoid arthritis	(70)
OLT1177^#^	NLRP3 ATPase	Blocks NLRP3 ATPase activity, restricts inflammasome activation	Osteoarthritis	(71)
AMG108	IL-1R1	Human monoclonal IL-1R1-antibody	Osteoarthritis	(72)

^*^approved drug but not for NLRP3; ^#^ongoing clinical trials.

All IL-1 inhibiting strategies are well tolerated in the majority of patients. The most common adverse effect is a dose-dependent skin irritation at the injection site. However, a substantially increased incidence of bacterial infections of the respiratory tract caused by Gram-positive bacteria, including pneumococci, *Staphylococcus aureus* (SA), and/or group A streptococci (GAS) are reported (77). Furthermore, IL-1 inhibiting therapies were associated with a higher incidence of fatal infections as compared to the placebo treated group. Therefore, treatment with IL-1 inhibiting drugs is not recommended for patients with an ongoing infection or with a history of severe infections (59, 77, 78).

## Role of NLRP3 Inflammasome in Pneumococcal Infections

Pneumococci, SA, and GAS are frequent colonizers of the upper respiratory tract (79). Colonization is usually asymptomatic in healthy individuals. However, imbalances in the immune system can lead to severe, invasive and even life-threatening diseases such as pneumonia and sepsis. The occurrence of the more severe forms of infection is commonly found in children younger than 5 years of age, elderly, and immuno-compromised population (80). Due to the immunosuppressive nature of IL-1 inhibiting agents, patients undergoing treatment seem to be at higher risk to develop infections caused by these bacteria (77, 81). In general, inflammation plays a crucial role in infectious diseases. Impaired or insufficient inflammatory response can result in prolonged and/or recurrent infections. In contrast, excessive hyper-inflammation is associated with fatal outcome (82, 83).

Pneumococci colonize the nasopharyngeal cavity of 20%–50% of children and 8%–30% of adults. They have been implicated as the most common etiologic agent of community-acquired pneumonia (80, 84). However, only limited number of studies investigated the role of inflammasome in pneumococcal infections and contrary results are reported. For example, one murine model study reported that *NLRP3^−/−^* mice are more susceptible to pneumococcal pneumonia (85). In contrast, a study on pneumococcal meningitis showed that mice with an active NLRP3 signaling have higher clinical scores, suggesting that NLRP3 activation contributes to brain injury (86). Since the incidence of respiratory tract infections is elevated in patient receiving IL-1 inhibiting agent, we will solely focus on the role of NLRP3 in respiratory pneumococcal infections.

Two of the most important secreted pneumococcal virulence determinants are hydrogen peroxide (H_2_O_2_) and the cholesterol-dependent cytolysin, pneumolysin (PLY) (87). Both factors are implicated in inflammasome activating and suppressive processes. Based on the pneumococcal serotypes used for the infection, the NLRP3-dependent IL-1β secretion by human cells varies. Macrophages infected with serotypes that are associated with invasive diseases and express low/non-hemolytic PLY (serotypes 1, 7F and 8), release lower amounts of IL-1β as compared to macrophages infected with serotypes expressing a fully active PLY (serotypes 2, 3, 6B, 9N) (88, 89). Being poor activators of the inflammasome, the invasive serotypes are potentially less efficiently recognized by the innate immune system and therefore, are less susceptible to immuno-mediated clearance. However, the exact mechanism of NLRP3 activation by PLY is unknown. This process is most likely of indirect nature (**Figure 1**). Studies on human neutrophils have shown that PLY-mediated NLRP3 activation is a result of potassium ion (K^+^) efflux. Experimental inhibition of K^+^ efflux in neutrophils resulted in impaired caspase-1 activation and subsequently in diminished IL-1β processing (**Figure 1**). Furthermore, it was shown that lysosomal destabilization did not play a role in PLY-mediated IL-1β processing in neutrophils (90). In general, IL-1β induces the production of chemoattractants, such as CXCL1 and CXCL2 by lung epithelial cells, which enhance neutrophil influx (91) and subsequent bacterial clearance at the site of infection (92, 93). Studies on pneumococcal infections of mouse peritoneal neutrophils indicate that NLRP3 inflammasome is mainly responsible for IL-1β secretion, while the AIM2 and NLRC4 inflammasomes are dispensable in these type of immune cells (94). Furthermore, neutrophil-derived IL-1β is involved in activation of Th17 cells. Th17-derived IL-17A acts as an additional chemoattractant-stimulating agent (95, 96) and indirectly mediates neutrophilia in the infected organs (**Figure 1**) (97). Nonetheless, neutrophil influx alone is not sufficient to clear pneumococci and macrophage influx is essential to ensure bacterial elimination (93, 98). A study by Hoegen and colleagues demonstrated that PLY was a key inducer of NRLP3 inflammasome and IL-1β expression in human differentiated THP-1 cells (86). In contrast to neutrophils, NLRP3 inflammasome activity was dependent on lysosomal destabilization, release of ATP, and cathepsin B activation (86). Furthermore, NLRP3 inflammasome activating synergistic effects of PLY and TLR agonists in dendritic cells and macrophages are reported (85, 88). However, this is rather a general inflammasome activating/priming effect. Apart from NLRP3, PLY can also activate AIM2 inflammasomes (99).

In contrast to PLY, only one study investigated the pneumococci-derived H_2_O_2_ and inflammasome interplay. By utilizing mouse bone marrow-derived macrophages (mBMDMs), Ertmann and Gekara have shown that mBMDMs infected with pneumococci accumulate large amounts of pro-IL-1β and procaspase-1 (100). Detection of the processed forms of IL-1β and caspase-1 was highly delayed and remained undetectable until 12 h post infection. However, the ratio of processed IL-1β and caspase-1 to their precursors was still very low (100). In contrast, *spxB*-mutant strain, which lacked H_2_O_2_ production, showed an intrinsically increased capacity to activate the inflammasome. The authors suggested that pneumococci employ H_2_O_2_-mediated inflammasome inhibition as a colonization strategy (100). Although the study provides highly relevant new insights into pneumococci-host interplay, verification of these results in human host system is warranted. However, host factors upstream or downstream of NLRP3 inflammasome also play a crucial role in colonization and infection processes. Studies in aged mice have suggested that an increase in endoplasmic reticulum stress and enhanced unfolded protein responses contribute to diminished assembly and activation of the NLRP3 resulting in failed clearance of pneumococci (101). In support of this, Krone and colleagues demonstrated that aged mice shows a delayed clearance of pneumococci in the nasopharynx as compared to young mice (102). The authors attributed the observed phenotype to the impaired innate mucosal immune responses in aged mice, including NLRP3 and IL-1β suppression. Furthermore, Lemon and colleagues demonstrated a prolonged colonization of *IL1R^-/-^* adult mice as compared to wild-type mice (93). The prolonged colonization was linked to reduced numbers of neutrophils at early stages of infection and reduced macrophage influx at later time points of carriage in *IL1R^-/-^* mice (93).

Apart from the bacterial pore-forming toxins, microbial RNA has also been implicated as a direct NLRP3 inflammasome activator (19). Studies showed that even small fragments of staphylococcal or group B streptococcal (GBS) RNA are sufficient for inflammasome activation in human THP-1-derived and mouse macrophages (103, 104). Based on detailed analyses of GBS and mouse macrophages interplay, Gupta and colleagues proposed that bacteria-mediated activation of NLRP3 inflammasomes requires bacterial uptake, phagolysosomal acidification, and toxin-mediated leakage. Subsequently, the free accessible bacterial RNA interacts with NLRP3 and activates the inflammasome cascade (104). Whether such mechanism applies to pneumococcal infections, remains to be elucidated.

In general, tightly controlled inflammasome activation in pneumococcal pneumonia is one of many important host defense mechanisms contributing to bacterial clearance (56). However, excessive NLRP3 activation can also lead to uncontrolled pyroptosis. The disproportionate gasdermin-D mediated cell membrane rupture in a variety of lung cells may result in a release of plethora of alarmins, including processed antigens, ATP, HMGB1, reactive oxygen species, cytokines, and chemokines (105). These prompt an immediate reaction from resident and recruited immune cells leading to a pyroptotic chain reaction with subsequent excessive tissue pathology (106). Furthermore, pathogen-associated antigens might disseminate to other organs resulting in a severe systemic hyper-inflammatory response (105). Whether such actions apply to pneumococcal pneumonia remains to be shown.

## Conclusion

Besides the crucial role of NLRP3 inflammasome activation in inflammation, many studies implicate NLRP3 inflammasome in the pathology of several autoimmune and auto-inflammatory disorders. Currently, the most common therapy for such diseases involves the use of immuno-suppressive, cytokine inhibiting therapies, such as IL-1 inhibitors. The immuno-suppression of patients by these agents results in side effects that often include respiratory tract infections caused by pneumococci. However, only a limited number of studies investigated the role of the NLRP3 inflammasome in pneumococcal respiratory infections. Future studies, especially those considering the complex interplay of human genetics, immuno-suppressive status, and age with pneumococcal colonization are needed to better understand the role of NLRP3 inflammasome in such infections.

## Author Contributions

SS and NS conceived the concept for this review article. SS, FC, and NS wrote the manuscript. All authors contributed to the article and approved the submitted version.

## Funding

This research is supported by the Federal Excellence Initiative of Mecklenburg Western Pomerania and European Social Fund (ESF) Grant KoInfekt (ESF_14-BM-A55-0001_16).

## Conflict of Interest

The authors declare that the research was conducted in the absence of any commercial or financial relationships that could be construed as a potential conflict of interest.
